# Supplementary material for: Functional impact and molecular binding modes of drugs that target the PI3K isoform p110δ
Source: Commun Biol. 2023 Jun 5;6:603. doi: 10.1038/s42003-023-04921-z (PMC10241892; doi:10.1038/s42003-023-04921-z)
Supplement: Supplementary file 2 — Supplementary Information [file 42003_2023_4921_MOESM2_ESM.pdf]

Supplementary Information

Functional impact and molecular binding modes of drugs that target the PI3K isoform p110δ

Floyd Hassenrück [1,2,3], Maria Farina-Morillas [1,2,3], Lars Neumann[1,2,3], Francesco Landini [1,2,3], Stuart Blakemore [1,2,3], Mina Rabipour [1,2,3], Juan Raul Alvarez-Idaboy [4], Christian P. Pallasch [1,2,3], Michael Hallek [1,2,3], Rocio Rebollido-Rios [1,2,3]*, Günter Krause [1,2,3]*

1 University of Cologne, Faculty of Medicine and Cologne University Hospital, Department I of Internal Medicine; Center for Integrated Oncology Aachen, Bonn, Cologne, Düsseldorf; Cologne, Germany

2 CECAD Cologne Cluster of Excellence on Cellular Stress Responses in Aging-Associated Diseases; Cologne, Germany

3 Center for Molecular Medicine Cologne, Cologne, Germany

4 Facultad de Química, Departamento de Física y Química Teórica, Universidad Nacional Autónoma de México, Mexico City, Mexico

Table of contents

Supplementary Figures **3**

Suppl. Fig. 1: Oncogenic potential of wt and mutant p110δ. 3

Suppl. Fig. 2: Meta-analysis of PI3Ki isoform selectivity 4

Suppl. Fig. 3: PI3Ki potencies in biochemical and cellular assay systems 5

Suppl. Fig. 4: Cytotoxicity of control substances 6

Suppl. Fig. 5: Expression of p110δ variants in malignant B cell lines 7

Suppl. Fig. 6: AKT phosphorylation in the presence of idelalisib 8

Suppl. Fig. 7: Idelalisib sensitivity of AKT phosphorylation in different cell lines 9

Suppl. Fig. 8: Functional involvement of PI3Kδ in cell functions of malignant B cells 11

Suppl. Fig. 9: PI3Ki resistance of PI3K isoform-dependent BaF3 cells owing to resistance mutations 13

Suppl. Fig. 10: Apo and holo states of p110δ along with idelalisib-specific molecular interactions 14

Suppl. Fig. 11: Molecular interactions of ZSTK474 with p110δ 16

Suppl. Fig. 12: Uncropped Western Blots 18

Suppl. Fig. 13: Time course controls of MD simulations 20

Supplementary Tables **21**

Suppl. Table 1: Classification of structurally diverse PI3Ki according to isoform selectivity 21

Suppl. Table 2: Potencies of PI3Ki determined in isogenic BaF3 cells 22

Suppl. Table 3: Potencies of PI3Ki in BaF3 cells carrying resistance mutations 23

Suppl. Table 4: Connecting PI3Ki resistance with reported binding pocket structures 24

Suppl. Table 5: Overview of performed molecular dynamis simulations 25

Suppl. Table 6: Oligonucleotide sequences 26

Suppl. Table 7: Antibodies used for signaling analyses 28

Supplementary References (referring to Supplementary Tables 1 and 4) **29**

Supplementary Figures

Supplementary Fig. 1 (related to *Fig. 1*)

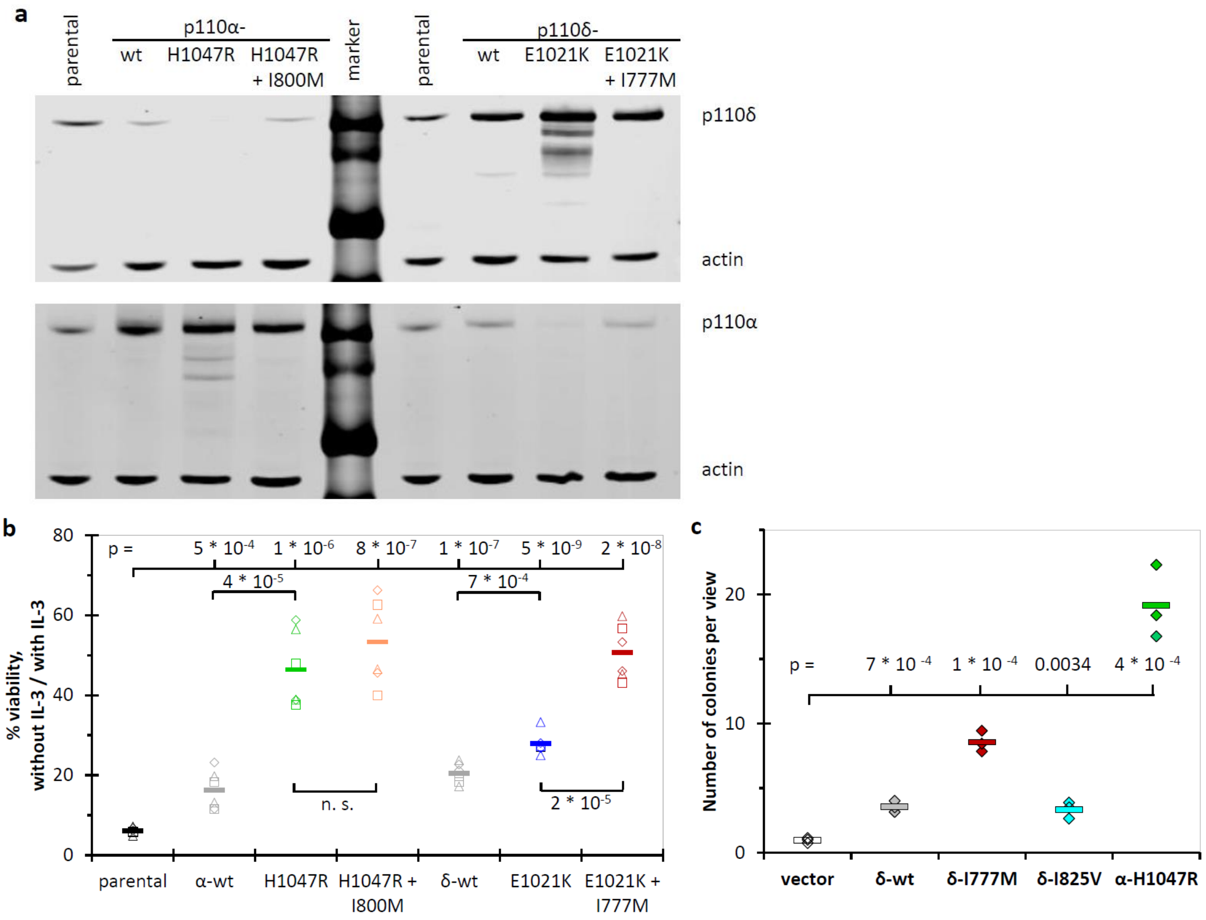

Supplementary Fig. 1: **Oncogenic potential of wt and mutant p110δ. (a)** Lysates of retrovirally transduced BaF3 cells were analyzed by immunoblotting for expression of p110δ (upper panel) or p110α (lower panel). Actin served as a loading control. **(b)** After selection by vector-encoded geneticin resistance, transduced BaF3 cells were examined for IL-3-independence in the CTG assay following three days of culture on 96-well plates with concomitant IL-3 withdrawal. Viability is expressed as percentages of the corresponding IL-3-complemented BaF3 cells. The results of two independent experiments with triplicate samples are shown. All p-values were determined in two-sided unpaired T-tests. **(c)** The anchorage-independent growth of NIH3T3 cells expressing wt or mutant p110-δ was assessed as their capacity to form colonies in soft agar and evaluated by microphotography and image analysis in 3 viewing areas of 1784.7 x 1328.7 µm^2 per condition. Numbers of colonies with an area of at least 1200 µm^2 were enumerated in three independent experiments.

Supplementary Fig. 2 (related to *Fig. 2*)

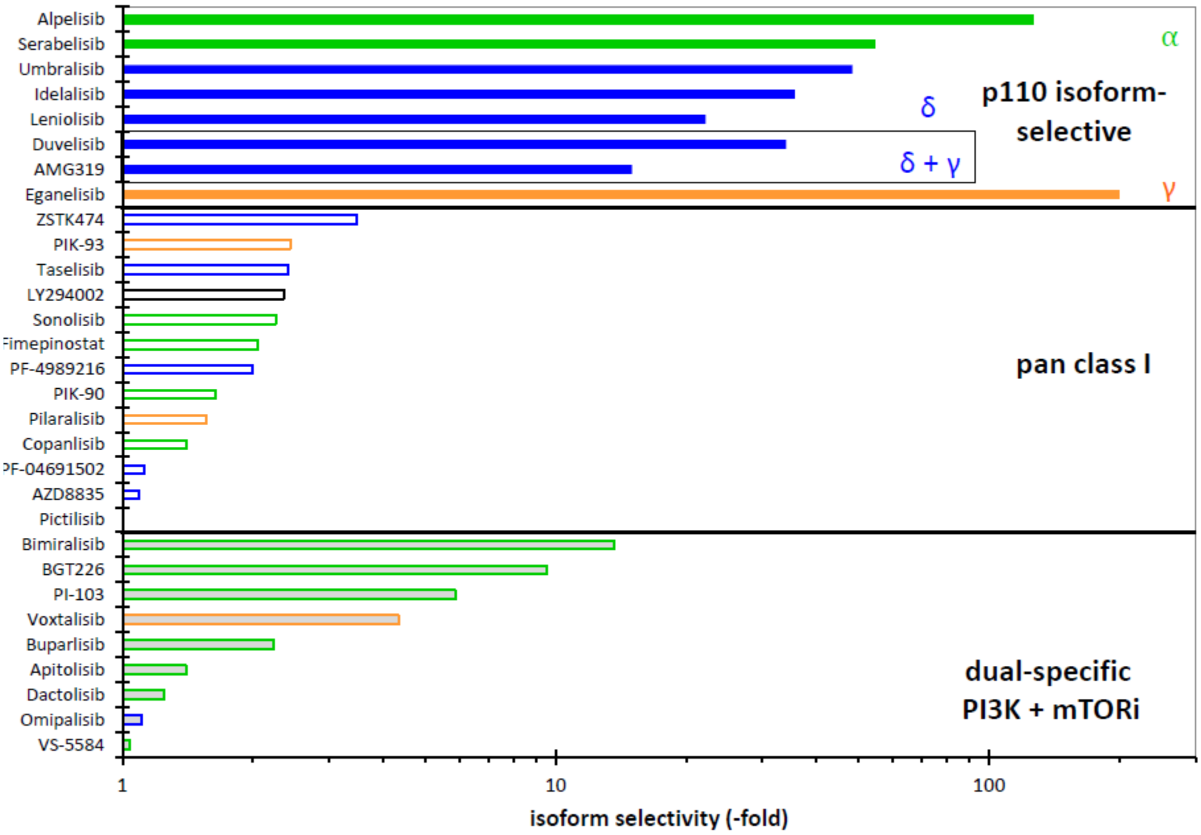

Supplementary Fig. 2: **Meta-analysis of PI3Ki isoform selectivity.** According to published inhibition of purified kinases, the investigated PI3Ki were assigned to isoform-selective PI3Ki and pan class I PI3Ki, which were subdivided according to additional activity against mTOR (*supplementary Table 1*). Within each category, PI3Ki are listed in the order of descending isoform selectivity. Colors indicate preferentially targeted isoforms (green: p110α, blue: p110δ, orange: p110γ).

Suppl. Fig. 3 (related to *Fig. 2*)

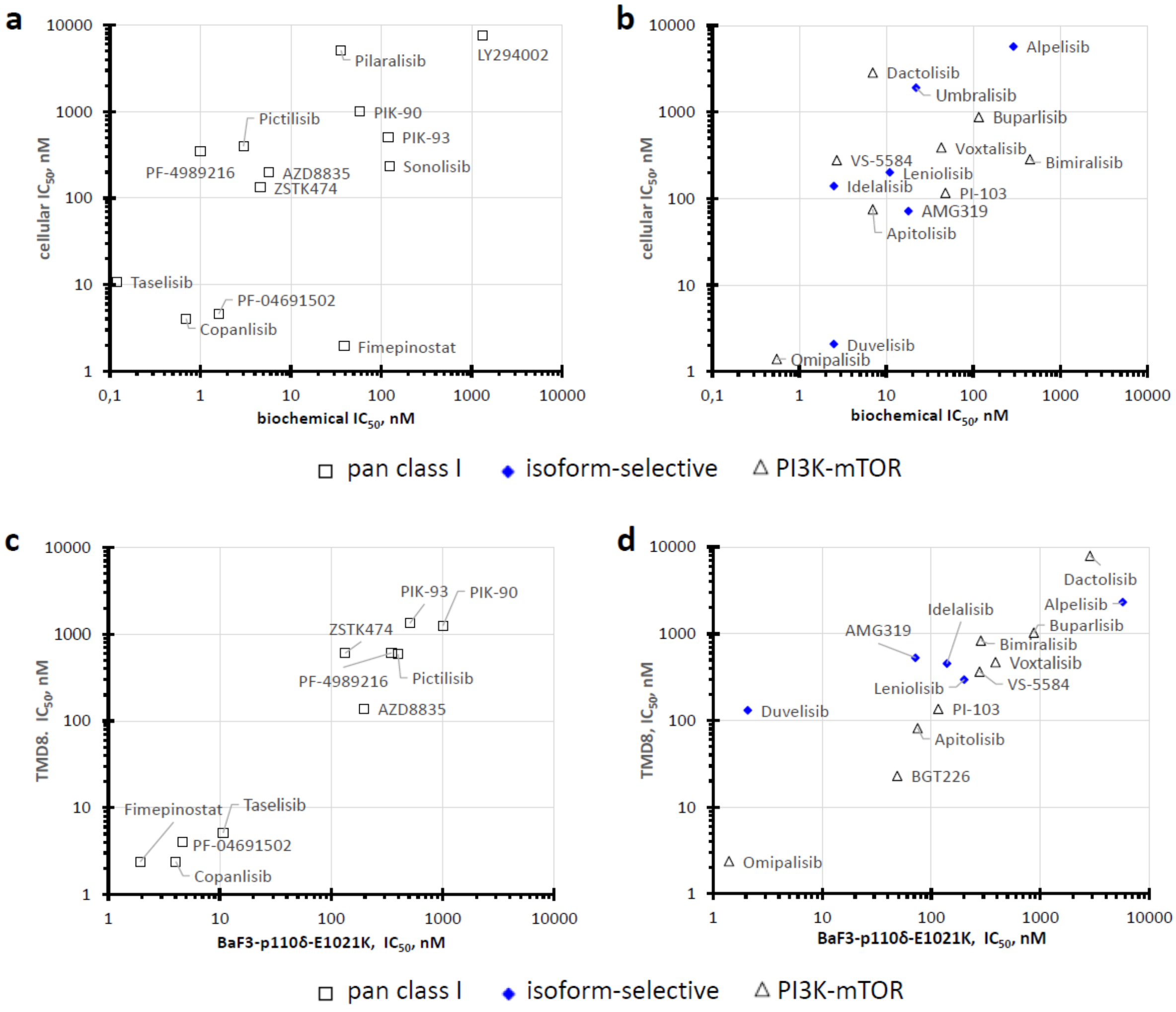

Supplementary Figure 3: **PI3Ki potencies in biochemical and cellular assay systems. (a, b)** The cellular PI3Ki potencies obtained with p110δ-dependent BaF3 cells were plotted against published biochemical potencies measured with purified p110δ (*supplementary Table 1*). **(c, d)** In a similar manner, PI3Ki potencies in the p110δ-selective mechanistic model were compared with those obtained with the DLBCL cell line TMD8. Results are shown in separate diagrams for pan class I PI3Ki **(a, c)** and isoform-selective PI3Ki as well as dual PI3K-mTOR inhibitors **(b, d)**.

*Suppl. Fig. 4 (*Related to *Figs. 2* and *5*)

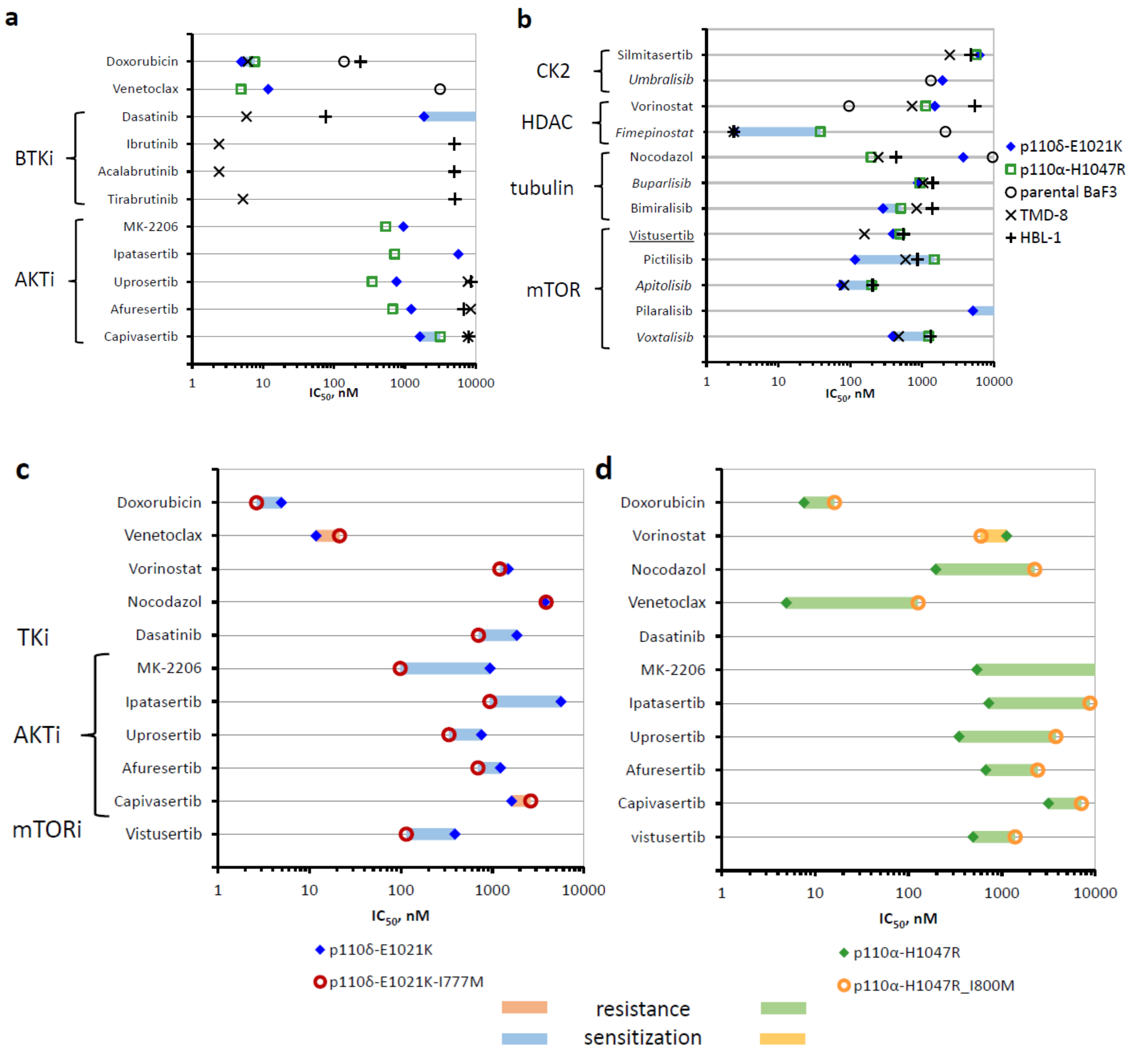

Supplementary Figure 4: **Cytotoxicity of control substances.** The cytotoxicity of control compounds and some PI3Ki was assessed in concentration-dependent CellTitre-Glo viability assays with isogenic BaF3 cells and the DLBCL cell lines TMD8 and HBl-1 on 384-well plates. **(a)** IC_{50} values obtained in five cell lines were compared for compounds with other mechanisms of cytotoxicity than PI3K inhibition. **(b)** Compounds with different mechanisms (underlined) were compared with PI3Ki known to inhibit additional target molecules (italics) and structurally related PI3Ki. Umbralisib was tested only in BaF3 cells expressing p110δ-E1021K on 96-well plates (*Supplementary Table 2*). **(c, d)** In addition the effect of resistance mutations on the cytotoxicity of control substances was examined. Resistance and sensitization owing to these affinity pocket mutations are indicated by blue and red bars, respectively, for p110δ **(c)** and by orange and green bars, respectively, for p110α **(d)**.

Supplementary Figure 5 (related to *Fig. 3*)

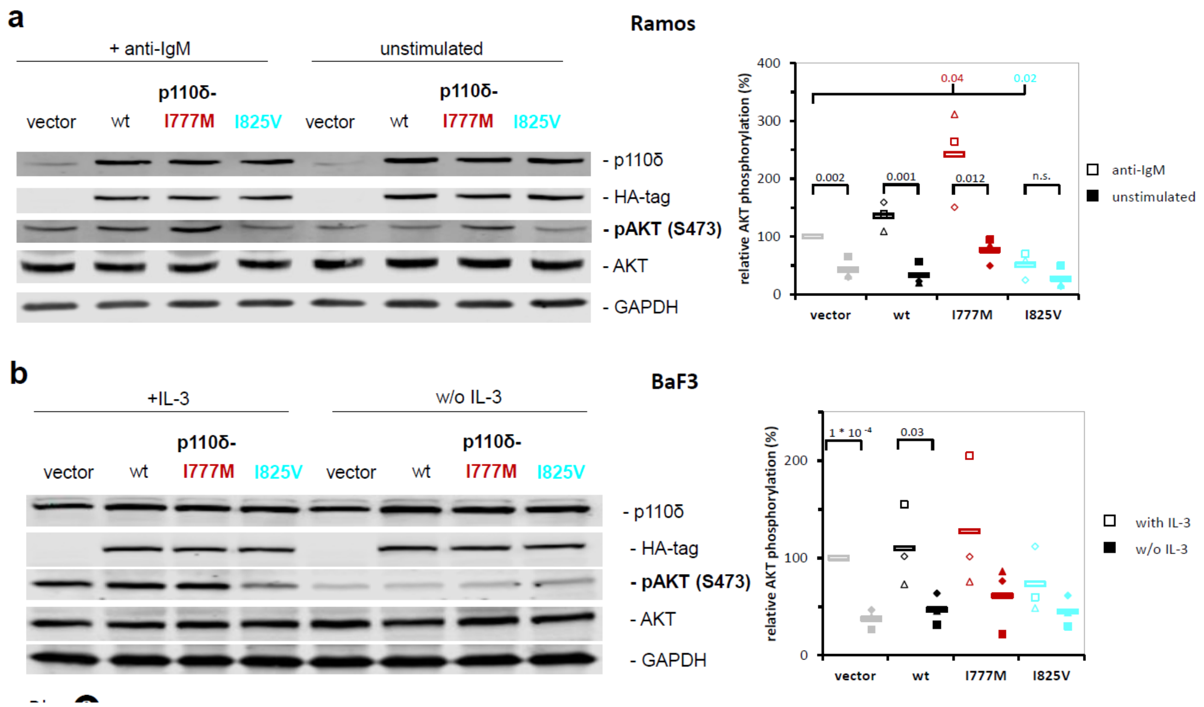

Supplementary Figure 5: **Expression of p110δ variants in malignant B cell lines.** Ramos **(a)** or BaF3 **(b)** cells were retrovirally transduced to express wt or mutant p110δ. Prior to lysis, serum-deprived cells were incubated without activation or after stimulation with anti-IgM **(a)** or IL-3 **(b)**. Overexpression of p110δ and its HA-tag proved successful retroviral transduction. In addition, the pAKT signals determined in immune blots were quantified and normalized to total AKT and the GAPDH loading control. The blots shown are representative of three independent experiments. Means and single values obtained in activated and unstimulated cells from three biological replicates were compared by unpaired two-sided T-test.

In contrast to the I777M substitution, the gatekeeper mutation I825V reduced pAKT levels compared to wt.

Supplementary Fig. 6 (related to *Fig. 3*)

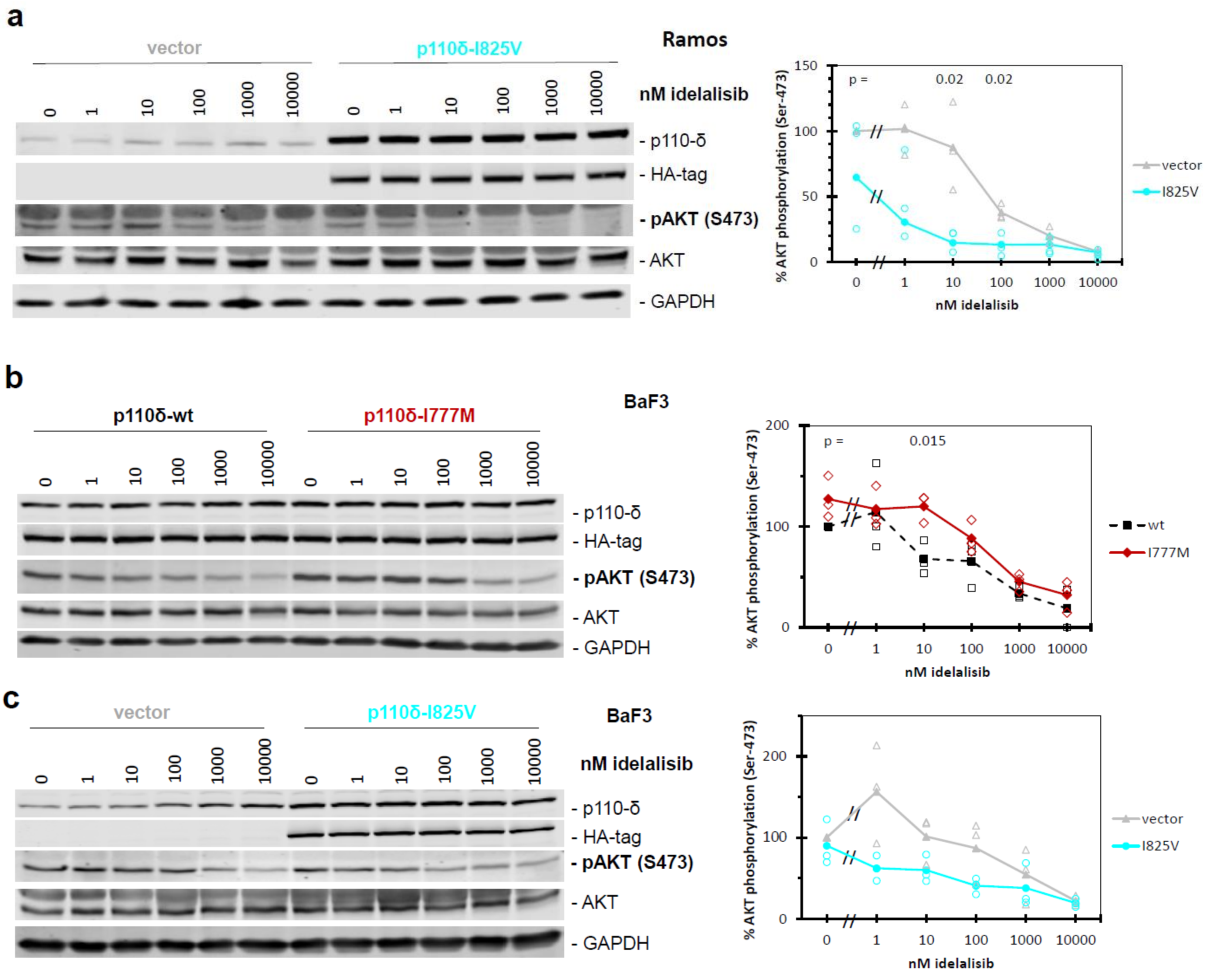

Supplementary Fig. 6: **AKT phosphorylation in the presence of idelalisib.** Concentration-dependent intracellular pAKT levels of anti-IgM-stimulated Ramos cells **(a)** and IL-3-stimulated BaF3 cells **(b, c)** that express wt or mutant p110δ were determined by immune blots. The Western blots shown are representative of three independent experiments that also served for quantitation. Fluorescence signals were normalized to the corresponding total AKT and GAPDH references and expressed as percentages of the untreated wild-type or vector controls, respectively. Means and single values obtained in activated and unstimulated cells from three biological replicates were compared by unpaired two-sided T-test.

While the I777M mutation led to gain-of-function and idelalisib resistance, the gatekeeper mutation I825V impaired p110δ-activity and mediated sensitization to idelalisib.

Supplementary Fig. 7 (related to *Fig. 3*)

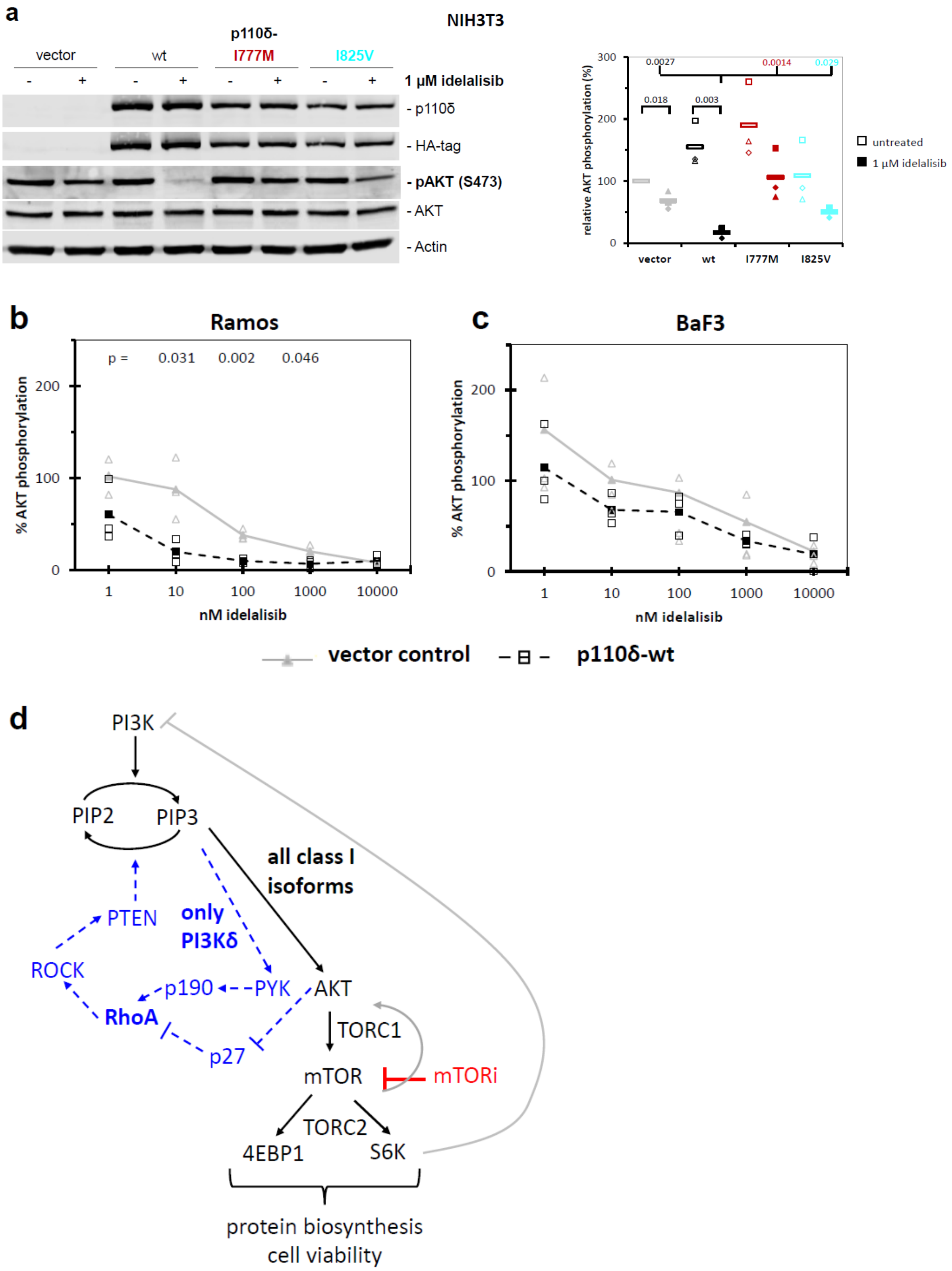

Supplementary Fig. 7: **Idelalisib sensitivity of AKT phosphorylation in different cell lines. (a)** NIH3T3 cells were transduced with vector alone or to stably express p110δ and subsequently treated with idelalisib. The pAKT signals in immune blots were quantified and normalized to total AKT and the GAPDH loading control. AKT phosphorylation was determined in three independent experiments and normalized to untreated vector control. **(b, c)** After idelalisib treatment, the AKT phosphorylation in Ramos **(b)** and BaF3 cells **(c)** was normalized to the respective untreated cell type. Vector controls and cells expressing p110δ were compared in two-sided unpaired T-tests. Endogenous p110δ expression increased from NIH3T3 cells **(a)** to Ramos and BaF3 cells (S*upplementary Figs. 6a* and *6b*) and transduced cells showed correspondingly decreasing overexpression of p110δ and sensitization to idelalisib. **(d)** Feedback loops in the PI3K/AKT signaling cascade. The scheme shows a p110δ-selective, negative feedback loop leading to PTEN activation (blue dashed lines) that can explain increased PI3Ki sensitivity upon p110δ overexpression, whereas most PI3K/AKT signaling (black) is mediated by all class I isoforms [37]. Concomitant pharmacological inhibition of mTOR (red) by dual-specific PI3K/mTORi therefore affects general PI3K/AKT signaling, while p110δ-selective PTEN activation owing to overexpression persists. In addition, TORC1- and TORC2-mediated positive feedback on AKT phosphorylation at serine 471 and negative feedback on targets upstream of PI3K are shown in grey.

Suppl. Fig. 8 (related to *Fig. 4*)

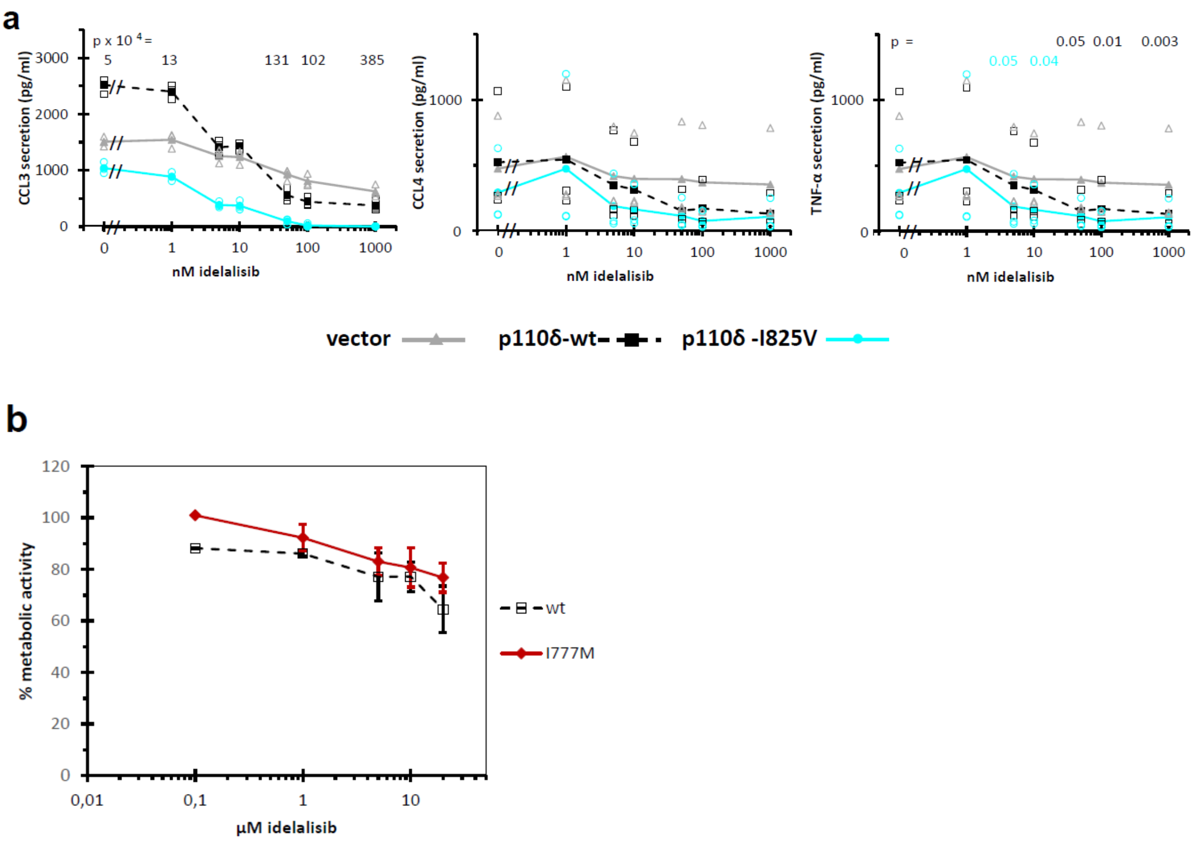

Supplementary Figure 8: **Functional involvement of PI3Kδ in cell functions of malignant B cells.** Expression and functionality of wt or mutant p110δ in transduced Ramos was confirmed by immune blotting. **(a)** For Ramos cells transduced with vector, p110δ-wt and p110δ-I825V, the anti-IgM induced secretion of the cytokines CCL3, CCL4 and TNFα in the presence of six concentrations of idelalisib was examined by ELISA of culture supernatants. Means and SEM of the percentages of cytokine secretion compared to untreated controls are shown. Three independent assays were performed. **(b)** Ramos cells expressing wt or mutant p110δ were cultured in the presence of the indicated concentrations of idelalisib for 48 hours. Subsequently their metabolic activity relative to untreated samples was determined in XTT assays.

Supplementary Fig. 9 (related to *Fig. 5*)

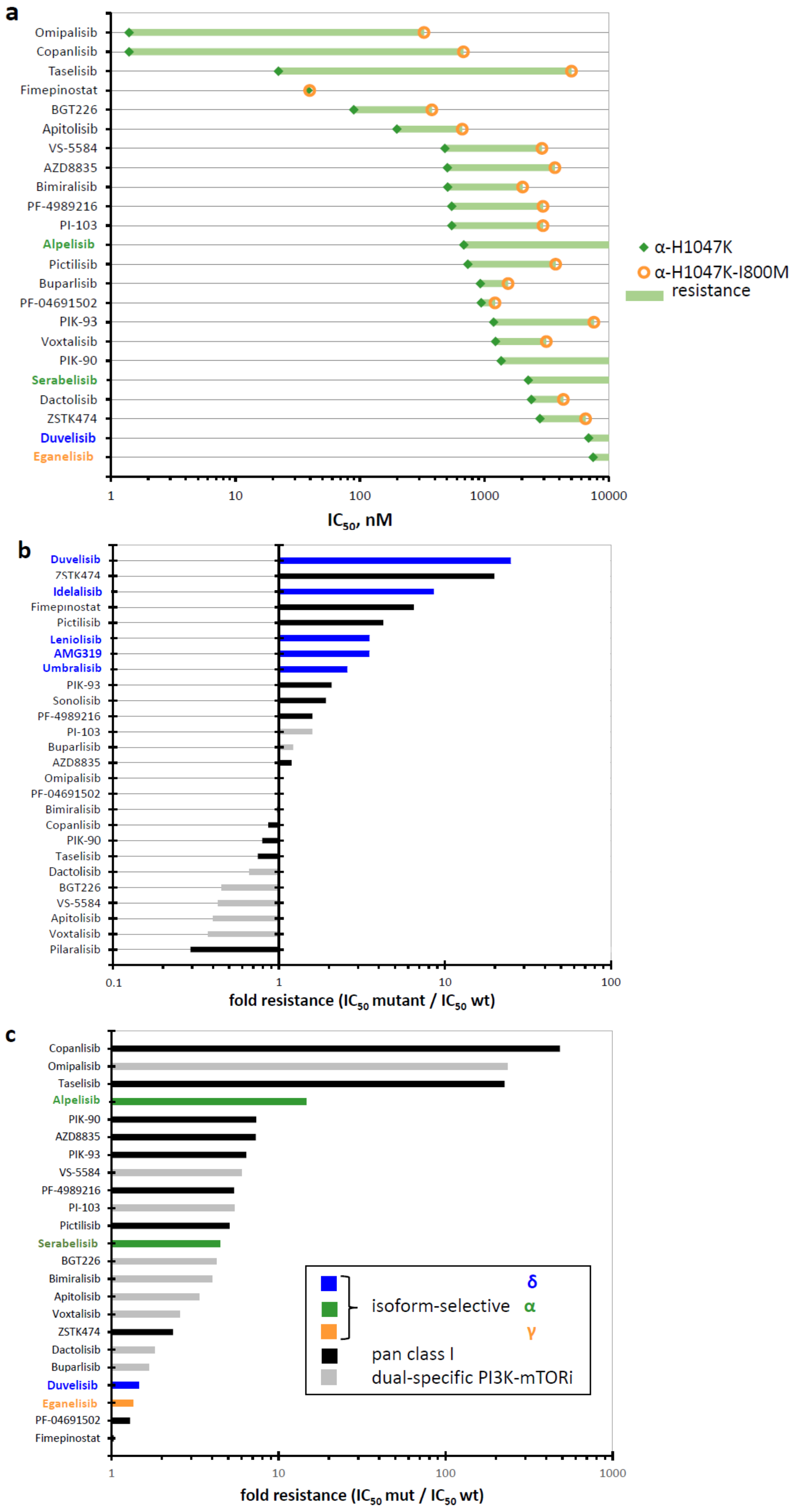

Supplementary Fig. 9: **PI3Ki resistance of PI3K isoform-dependent BaF3 cells owing to resistance mutations. (a)** PI3Ki that showed cellular IC_{50} below 10 µM with BaF3 cells expressing p110α-H1047R were arranged according to decreasing cellular IC_{50} potencies and compared with the corresponding IC_{50} values of isogenic cells with additional I800M mutation. Orange bars indicate resistance. **(b, c)** PI3Ki were ranked according to the resistance determined as ratios of cellular IC_{50} values (mutant/wt) obtained with BaF3 cells expressing p110δ-E1021K **(b)** or p110α-H1047R **(c)** without or with the corresponding resistance mutation I777M or I800M, respectively.

Supplementary Fig. 10 (related to *Fig. 6*)

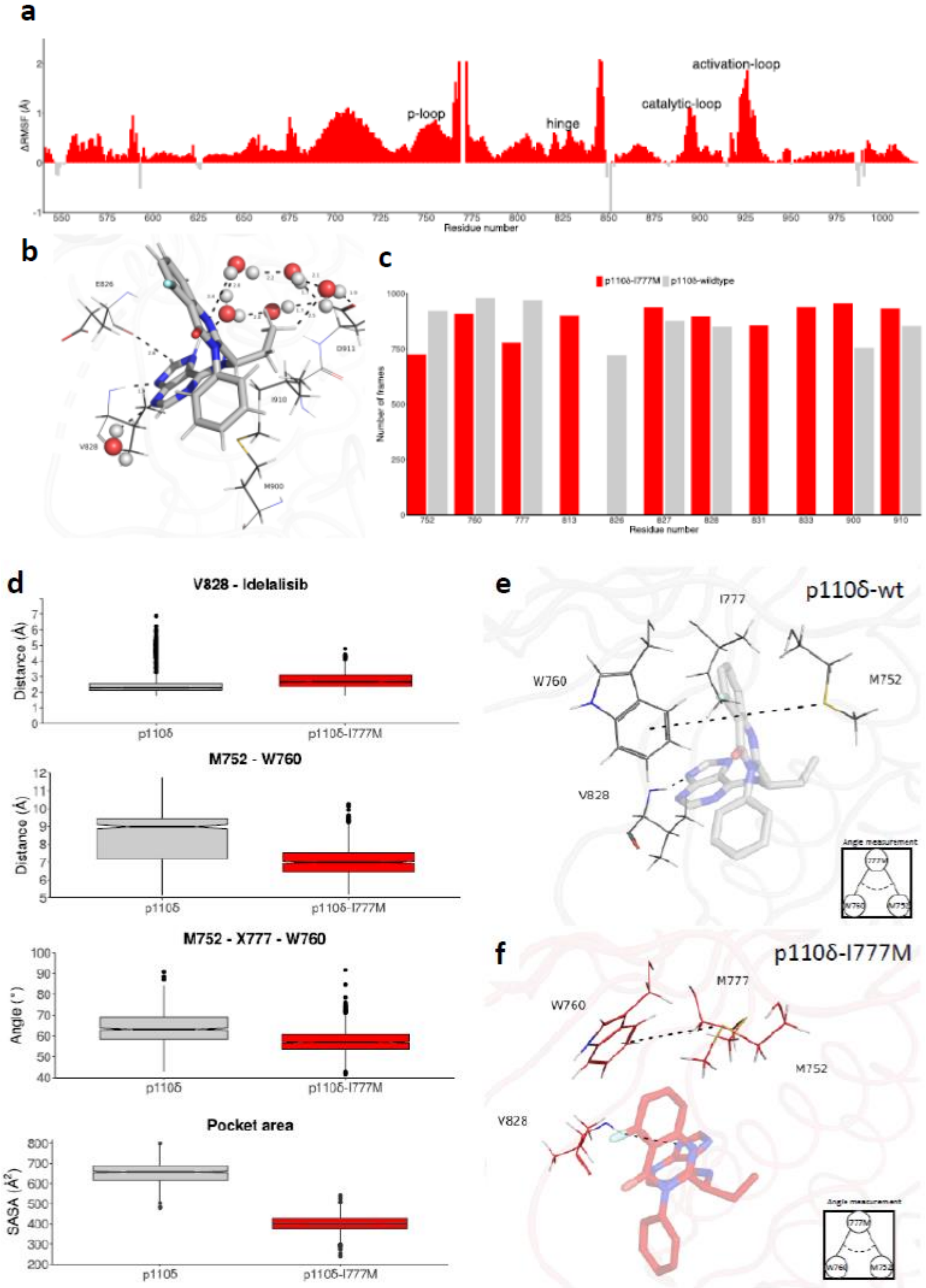

Specification of parameters	wt	I777M	p-value
Distance M752 SD atom - W760 COM benzene ring	8.96 Å	6.98 Å	$< 2.2 * 10^{-16}$
Distance V828 NH – idelalisib N31 atom	2.26 Å	2.66 Å	$< 2.2 * 10^{-16}$
Angle M752 SD atom – X777 CA atom – W760 COM benzene ring	63.35 °	57.11 °	$< 2.2 * 10^{-16}$
Solvent accessible surface area (SASA) of binding pocket	656.92 Å²	401.02 Å²	$< 2.2 * 10^{-16}$

Supplementary Fig. 10: **Apo and holo states of p110δ along with idelalisib-specific molecular interactions. (a)** The root mean square fluctuation (RMSF) of kinase domain backbone atoms was determined in p110δ apo-forms, i.e. without inhibitor binding. The differences in RMSFs (ΔRMSF) between p110δ-I777M and p110δ-wt are plotted against the amino acid sequence, in which important regions are labeled. Red or grey columns indicate higher RMSF and hence flexibility of mutant or wt p110δ, respectively. **(b)** Idelalisib binding in the p110δ-wt pocket is mediated by water molecules with an extended network of hydrogen bonds. A cluster representative structure derived from a 1 µs simulation is shown. **(c)** Residues within 3 Å to idelalisib in p110δ-wt (grey) or p110δ-I777M (red). The height of the bars represents the total number of frames, in which the specific residues were found. **(d-f)** Detailed structural features of idelalisib binding to p110δ-wt (grey) or p110δ-I777M (red) were calculated from MD simulations. Median values of measurements and exact p-values calculated using the Wilcoxon signed-rank test with a statistical significance of 0.05 are given in the bottom Table. The compared distances and angles are also indicated by dashed lines or inserts in structures of idelalisib bound to p110δ-wt **(e)** and p110δ-I777M **(f)** from representative structures.

Supplementary Fig. 11 (related to *Fig. 7*)

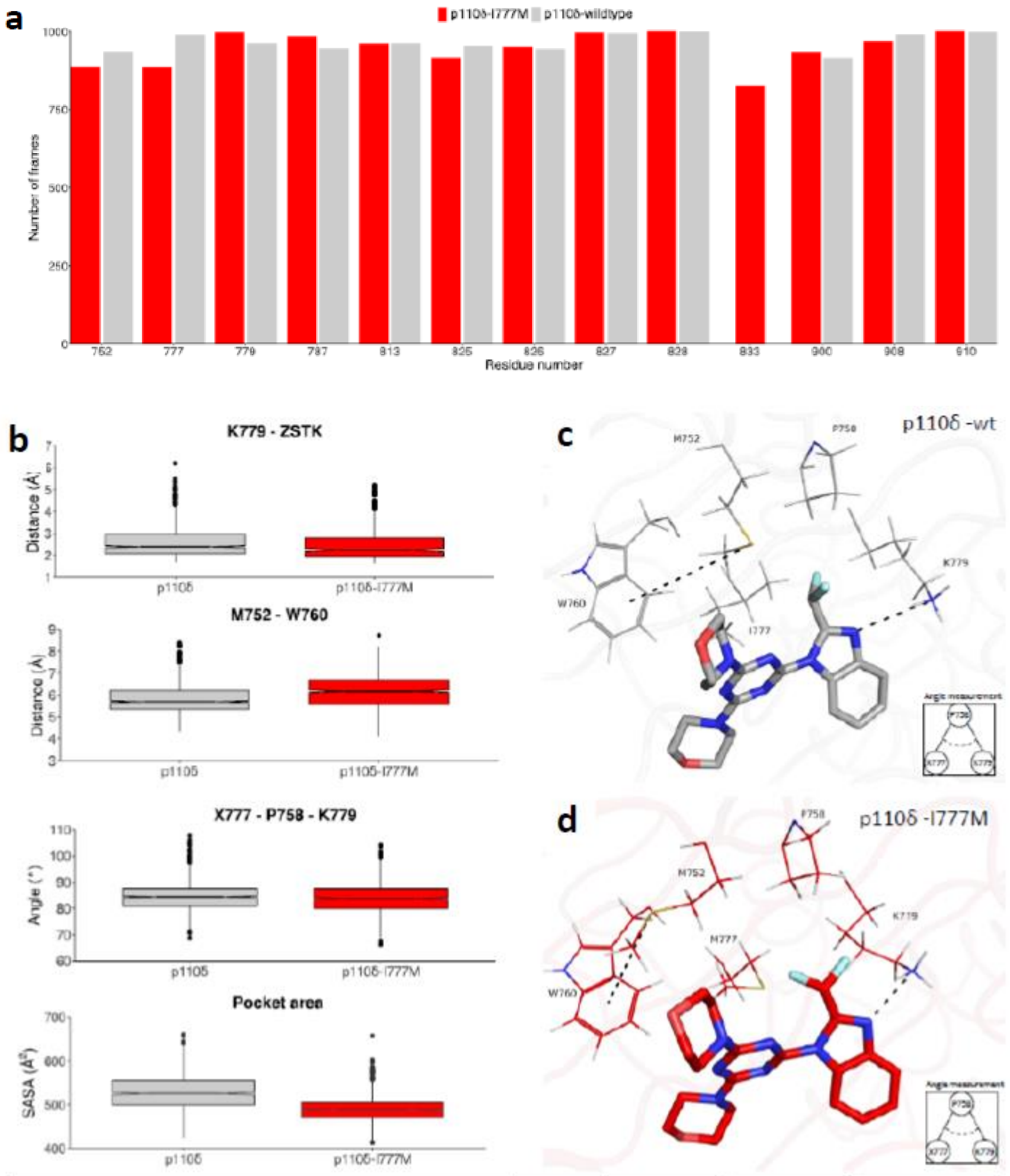

Specification of parameters	wt	I777M	p-value
Distance M752 SD atom - W760 COM benzene ring	5.69 Å	6.17 Å	$< 2.2 * 10^{-16}$
Distance K779 ZH – ZSTK474 N31 atoms	2.40 Å	1.92 Å	$< 2.2 * 10^{-16}$
Angle X777 CB – P758 CB – K779 NZ atoms	84.48 °	83.76 °	0.0096
Solvent accessible surface area (SASA) of binding pocket	525.27 Å²	486.90 Å²	$< 2.2 * 10^{-16}$

Supplementary Fig. 11: **Molecular interactions of ZSTK474 with p110δ. (a)** Residues within 3 Å to ZSTK474 in p110δ-wt (grey) and p110δ-I777M (red). The height of the bars represents the total number of frames, in which the specific residues were found. **(b-d)** Detailed structural features of ZSTK474 binding to wt and mutant p110δ were calculated from MD simulations. Median values of measurements and exact p-values calculated using the Wilcoxon signed-rank test with a statistical significance of 0.05 are given in the bottom Table. The compared distances and angles are also indicated by dashed lines or inserts in structures of ZSTK474 bound to p110δ-wt **(c)** and p110δ-I777M **(d)** from representative structures.

Suppl. Fig. 12 (related to Methods)

Fig. 3a

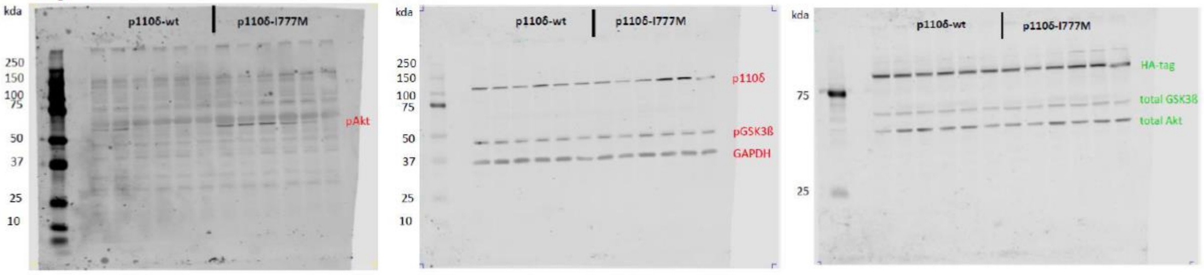

Suppl. Fig. 5a

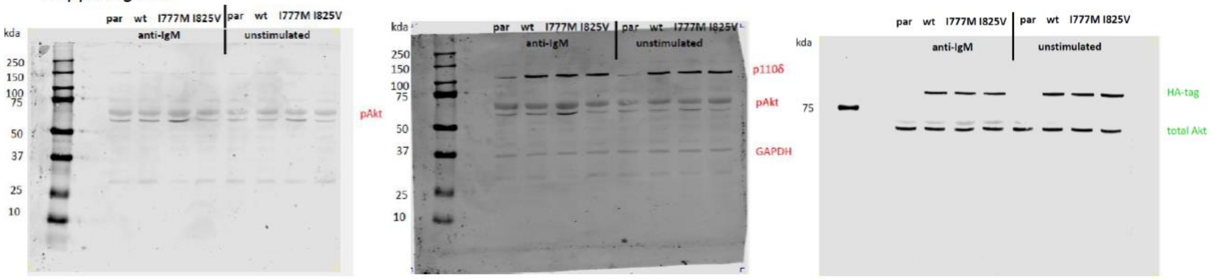

Suppl. Fig. 5b

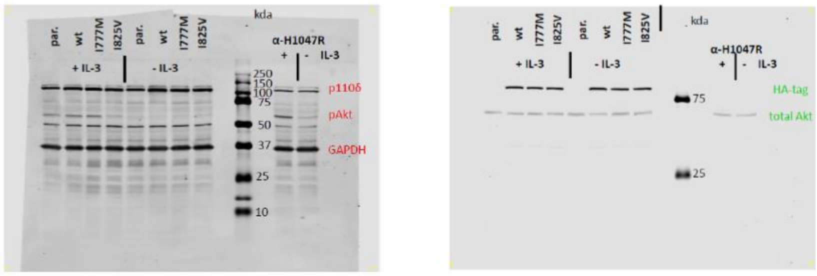

Suppl. Fig. 6a

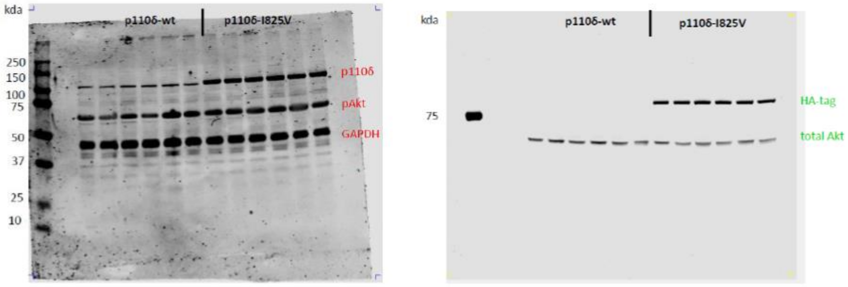

Suppl. Fig. 6b

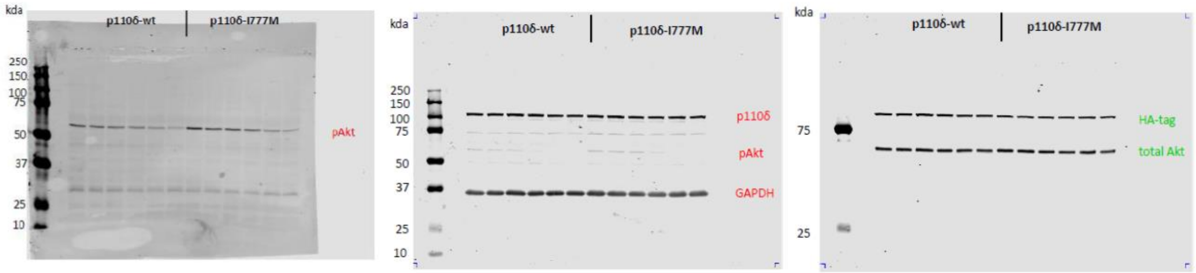

Suppl. Fig. 12 (continued)

Suppl. Fig. 6c

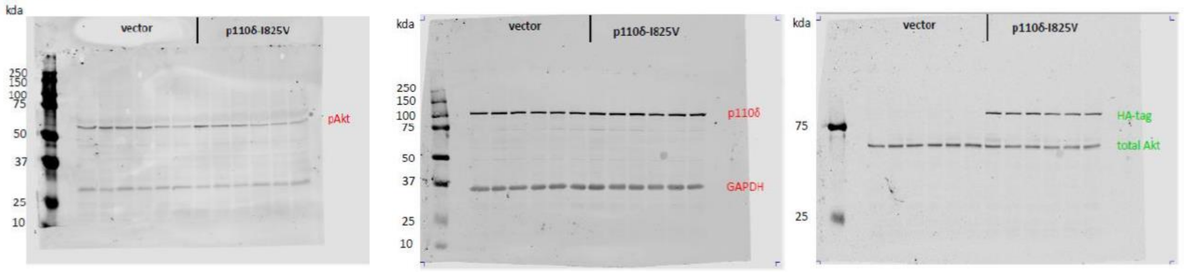

Suppl. Fig. 7a

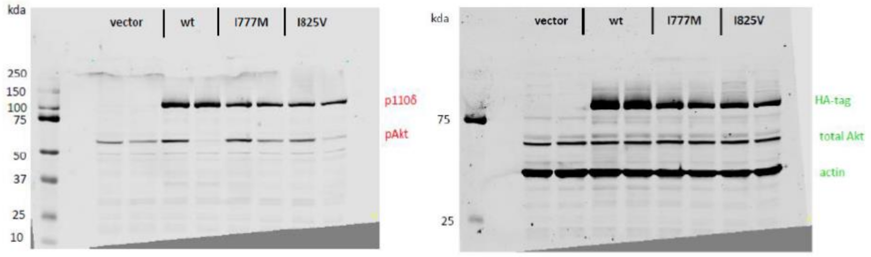

Supplementary Figure 12: **Uncropped Western Blots.** The provided whole gel scans served as sources for the mentioned main text Figures. They contain the sizes of molecular weight markers in kda. Bands detected with IR-dye680-labeled anti-rabbit or IR-dye 800-labeled anti-mouse secondary antibodies are indicated in red or green print, respectively.

Suppl. Fig. 13 (related to Methods)

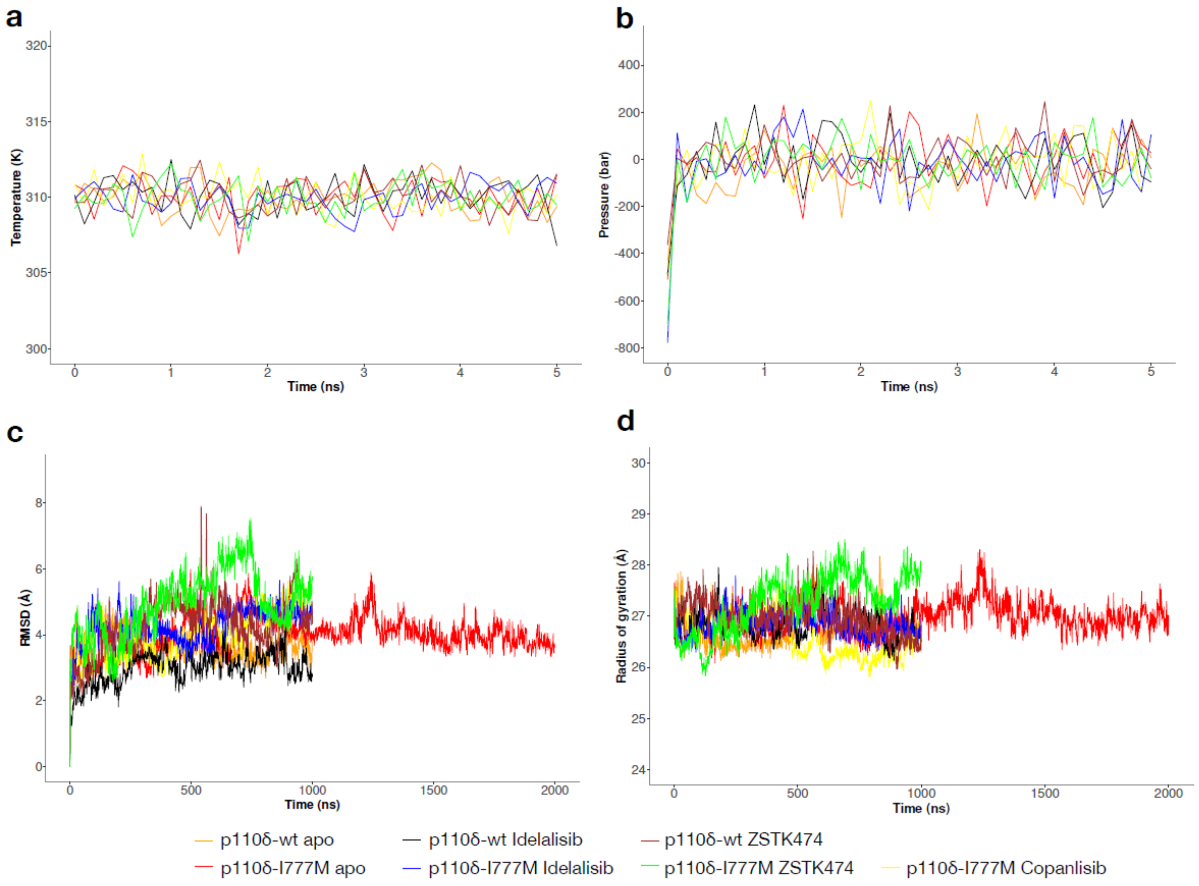

Supplementary Figure 13: **Time course controls of MD simulations. (a, b)** Convergence analyses of equilibrated MD simulations. **(a)** NVT ensemble with equilibrated temperature at 310 K. **(b)** NPT ensemble showing the pressure progression as a function of time for all the systems simulated. **(c, d)** Convergence analyses of production MD simulations. **(c)** Root-mean-square deviation (RMSD) of backbone atoms and **(d)** radius of gyration of the protein are plotted as a function of time for all the systems simulated.

Supplementary Tables

Supplementary Table 1: **Classification of structurally diverse PI3Ki according to isoform selectivity.**

Isoform-selective PI3Ki

No.	PI3Ki	Biochemical IC_{50} (nM)				Preferentially targeted isoform	Isoform selectivity *	Reference
		α	β	δ	γ			
1	Serabelisib	15	4500	13900	1900	α	126.7	1
2	Alpelisib	4.6	1156	290	250	α	54.3	2
3	Umbralisib	10000	1116	22	1065	δ	48.4	3
4	Idelalisib	820	565	2.5	89	δ	35.6	4
5	Leniolisib	244	424	11	2230	δ	22.2	5
6	Duvelisib	1602	85	2.5	27	δ + γ	34.0	6
7	AMG319	33000	270	18	85	δ + γ	15.0	7
8	TG100-115	1300	1200	235	83	γ + δ	14.5	8
9	Eganelisib	3200	3500	8400	16	γ	200.0	9
10	CZC24832	10000	1258	7943	25	γ	50.3	10
11	AS-604850	4500	20000	20000	250	γ	18.0	11

Pan class I PI3Ki

No.	PI3Ki	Biochemical IC_{50} (nM)				Preferentially targeted isoform	Isoform selectivity *	Reference
		α	β	δ	γ			
12	ZSTK474	16	44	4.6	49	δ	3.5	12
13	CH5132799	14	120	500	36	α	2.6	13
14	PIK-93	39	590	120	16	γ	2.4	14
15	Taselisib	0.29	91	0.12	0.97	δ	2.4	15
16	LY294002	720	306	1330	7260	β	2.4	11
17	Fimepinostat	19	54	39	311	α	2.1	16
18	PF-4989216	2	142	1	65	δ	2.0	17
19	PIK-90	11	350	58	18	α	1.6	14
20	Pilaralisib	39	383	36	23	γ	1.6	18
21	Copanlisib	0.5	3.7	0.7	6.4	α	1.4	19
22	PF-04691502	1.8	2.1	1.6	1.9	δ	1.1	20
23	AZD8835	6.2	431	5.7	90	δ	1.1	21
24	Pictilisib	3	33	3	75	δ	1.0	22

Dual PI3K/mTORi

No.	PI3Ki	Biochemical IC_{50} (nM)				Preferentially targeted isoform	Isoform selectivity *	Reference
		α	β	δ	γ			
25	Bimiralisib	33	661	451	708	α	13.7	23
26	BGT226	4	63	n.d.	38	α	9.5	24
27	PI-103	8.2	88	48	150	α	5.9	14
28	Voxtalisib	39	113	43	9	γ	4.3	25
29	Buparlisib	52	166	116	262	α	2.2	26
30	Apitolisib	5	27	7	14	α	1.4	27
31	Dactolisib	4	75	7	5	α	1.3	28
32	Omipalisib	0.61	1.8	0.55	1.2	δ	1.1	29
33	VS-5584	2.6	21	2.7	3	α	1.0	30

* Selectivity is expressed as the –fold excess over the next potently targeted isoform corresponding to the minimal among the calculated IC_{50} ratios. Within subclasses, inhibitors are listed in the order of descending isoform selectivity.

Supplementary Table 2: **Potencies of PI3Ki determined in isogenic BaF3 cells.**

Rank [a]	Inhibitor	Class	Cellular IC_{50} (nM) [b]			
			BaF3-p110-δ-E1021K		BaF3-p110-α-H1047R	
			median	MAD	median	MAD
1	Omipalisib	PI3K/mTOR	<2.4	n.a. [c]	2	n.a. [c]
2	Fimepinostat	pan class I	<2.4	n.a. [c]	38	11
3	Duvelisib	isoform-selective δ	2.6	n.a. [c]	6857	849
4	Copanlisib	pan class I	4.5	2	2	n.a. [c]
5	PF-04691502	pan class I	5.1	3	943	781
6	Taselisib	pan class I	11	7	22	17
7	BGT226	PI3K/mTOR	49	11	89	63
8	AMG319	isoform-selective δ	72	43	>10000	n.a. [c]
9	Apitolisib	PI3K/mTOR	75	14	198	33
10	Pictilisib	pan class I	116	70	1472	167
11	ZSTK474	pan class I	133	81	2784	297
12	Idelalisib	isoform-selective δ	139	87	>10000	n.a. [c]
	Idelalisib [d]		53	40	n.d. [c]	n.d. [c]
13	AZD8835	pan class I	197	97	504	21
14	Leniolisib	isoform-selective δ	201	99	>10000	n.a. [c]
15	VS-5584	PI3K/mTOR	278	48	481	296
16	Bimiralisib	PI3K/mTOR	285	160	507	160
17	PF-4989216	pan class I	348	278	546	212
18	Voxtalisib	PI3K/mTOR	390	182	1227	529
19	PI-103	pan class I	398	165	735	77
20	Sonolisib [d]	pan class I	491	256	n.d. [c]	n.d. [c]
21	PIK-93	pan class I	508	208	1181	755
22	Buparlisib	PI3K/mTOR	875	158	928	318
23	PIK-90	pan class I	1003	547	1362	138
24	Umbralisib [d]	isoform-selective δ	1881	522	n.d. [c]	n.d. [c]
25	Dactolisib	PI3K/mTOR	2868	1285	2383	724
26	Pilaralisib	pan class I	5081	2706	>10000	n.a. [c]
27	Alpelisib	isoform-selective α	5732	4278	682	544
28	Eganelisib	isoform-selective γ	7442	2559	7456	2545
29	LY294002	pan class I	7527	2474	>10000	n.a. [c]
30	Serabelisib	isoform-selective α	9278	723	2248	1262

[a]: Investigated PI3Ki were ranked according to descending cellular potency against BaF3 cells expressing p110δ-E1021K.

[b]: Determined in four or three independent assays for p110δ or p110α, respectively, with quadruplicate samples.

[c]: n.a.: not applicable; n.d.: not determined.

[d]: Measurements on 96-well plates.

Supplementary Table 3: **Potencies of PI3Ki in BaF3 cells carrying resistance mutations.**

**Rank** [a]	**Inhibitor**	**Class**	**Cellular IC_{50} (nM)** [b] **in BaF3 cells expressing**			
			**p110-δ-E1021K-I777M**		**p110-α-H1047R-I800M**	
			**median**	**MAD**	**median**	**MAD**
1	Omipalisib	PI3K/mTOR	<2.4	n.a. [c]	327	267
2	Fimepinostat	pan class I	13	11	39	2
3	Duvelisib	isoform-selective δ	52	19	>10000	n.a. [c]
4	Copanlisib	pan class I	4.0	1.6	677	276
5	PF-04691502	pan class I	4.6	1.8	1216	639
6	Taselisib	pan class I	8	3	5003	4998
7	BGT226	PI3K/mTOR	22	9	378	126
8	AMG319	isoform-selective δ	252	133	>10000	n.a. [c]
9	Apitolisib	PI3K/mTOR	30	9	663	269
10	Pictilisib	pan class I	495	354	6540	3461
11	ZSTK474	pan class I	2655	1074	6501	3360
12	Idelalisib	isoform-selective δ	1199	115	>10000	n.a. [c]
	Idelalisib [d]		634	264	n.d. [c]	n.d. [c]
13	AZD8835	pan class I	236	107	3682	310
14	Leniolisib	isoform-selective δ	707	199	>10000	n.a.
15	VS-5584	PI3K/mTOR	119	4	2895	344
16	Bimiralisib	PI3K/mTOR	277	231	2023	1804
17	PF-4989216	pan class I	555	530	2954	1263
18	Voxtalisib	PI3K/mTOR	146	83	3139	2817
19	PI-103	pan class I	571	349	3735	842
20	Sonolisib [d]	pan class I	946	243	n.d. [c]	n.d. [c]
21	PIK-93	pan class I	1057	330	7579	2422
22	Buparlisib	PI3K/mTOR	1067	282	1548	920
23	PIK-90	pan class I	796	323	>10000	n.a. [c]
24	Umbralisib [d]	isoform-selective δ	4866	1065	n.d. [c]	n.d. [c]
25	Dactolisib	PI3K/mTOR	1897	909	4307	1886
26	Pilaralisib	pan class I	1489	920	>10000	n.a. [c]
27	Alpelisib	isoform-selective α	>10000	n.a. [c]	>10000	n.a. [c]
28	Eganelisib	isoform-selective γ	>10000	n.a. [c]	>10000	n.a. [c]
29	LY294002	pan class I	1244	153	>10000	n.a. [c]
30	Serabelisib	isoform-selective α	>10000	n.a. [c]	>10000	n.a. [c]

[a]: The investigated PI3Ki were ranked according to descending potency against BaF3 cells expressing p110δ-E1021K as in supplementary Table S2.

[b]: Determined in four or three independent assays for p110δ or p110α, respectively, with quadruplicate samples.

[c]: n.a.: not applicable; n.d.: not determined.

[d]: Measurements on 96-well plates.

Supplementary Table 4: **Connecting PI3Ki resistance with reported binding pocket structures.**

Rank [a]	Inhibitor	Class	Fold resistance	substitute	PDB entry[b]	supplementary reference
1	**Duvelisib**	*isoform-sel. δ*	24.98	idelalisib	δ: 4XE0	[31]
2	ZSTK474	pan class I	19.92		δ: 2WXL	[32]
3	**Idelalisib**	*isoform-sel. δ*	8.60		δ: 4XE0	[32]
4	CUDC-907	pan class I	6.51		n.a. [c]	
5	Pictilisib	pan class I	4.26		δ: 2WXP	[32]
6	**Leniolisib**	*isoform-sel. δ*	3.52		δ: 5O83	[5]
7	**AMG319**	*isoform-sel. δ*	3.51	IC87114	δ: 2X38	[32]
8	**Umbralisib**	*isoform-sel. δ*	2.59	IC87114	δ: 2X38	[32]
9	PIK-93	pan class I	2.08		γ: 2CHZ	[14]
10	Sonolisib	pan class I	1.93	wortmannin	γ: 1E7U	[33]
11	*PI-103*	PI3K-mTOR	1.59		α: 4L23	[34]
12	PF-4989216	pan class I	1.59		n.a. [c]	
13	*Buparlisib*	PI3K-mTOR	1.22		γ: 1EAZ	[26]
14	AZD8835	pan class I	1.20		α: 2RD0 [d]	[21]
15	*Omipalisib*	PI3K-mTOR	1.00		γ: 3L08	[35]
16	PF-04691502	pan class I	1.00		γ: 3ML8/9	[36]
17	*Bimiralisib*	PI3K-mTOR	0.97		γ: 5OQ4	[23]
18	Copanlisib	pan class I	0.86		γ: 5G2N	[37]
19	PIK-90	pan class I	0.79		γ: 2CHX	[14]
20	Taselisib	pan class I	0.75		δ: 5T8F	[38]
21	*Dactolisib*	PI3K-mTOR	0.66		γ: 3SD5	[28]
22	*BGT226*	PI3K-mTOR	0.45		n.a. [c]	
23	*VS-5584*	PI3K-mTOR	0.43		n.a. [c]	
24	*Apitolisib*	PI3K-mTOR	0.40		γ: 3TL5	[27]
25	*Voxtalisib*	PI3K-mTOR	0.37		n.a. [c]	
26	Pilaralisib	pan class I	0.29		n.a. [c]	

[a]: Position in a ranking according to descending resistance mediated by p110δ-I777M (*Extended Data Fig. 6B*). PI3Ki with selectivity for p110δ are printed in blue; dual PI3K-mTOR inhibitors in italics.

[b]: Structural information from X-ray crystallography of co-crystals with the indicated p110 isoforms is accessible from the Research Collaboratory for Structural Bioinformatics Protein Data Bank (RSCB PDB).

[c]: 3D co-crystal structures not available (n.a.) for the respective PI3Ki or similar molecules as indicated in the column "substitute".

[d]: Apo-form structure, to which the AZD8835 molecule was docked.

Supplementary Table 5: **Overview of performed molecular dynamics simulations.**

ID	System [a]	Structure	Inhibitor name	Simulation time (µs)	Box volume (Å^3)	Total number of atoms	Total number of waters
1	p110δ-wt	apo	-	1	9949.1	97472	88560
2	p110δ-wt	holo	Idelalisib	1	11025.7	107789	98808
3	p110δ-wt	holo	ZSTK474	1	9948.3	97526	88563
4	p110δ-I777M	apo	-	2	10184.9	98527	89613
5	p110δ-I777M	holo	Idelalisib	1	10304.4	98722	89757
6	p110δ-I777M	holo	ZSTK474	1	10201.7	98608	89643
7	p110δ-I777M	holo	Copanlisib	1	10214.2	98680	89703

[a] : The MD simulations were conducted at a salt concentration of 0.15 M.

Supplementary Table 6: Oligonucleotide sequences.

E1021 substitution in p110δ [1]

Wild type sequence [2]	1021<br>N'.. Arg Val Lys Phe Asn **Glu** Ala Leu Arg Glu Ser ..C'<br>5'.. CGA GTG AAG TTT AAC **G**AA GCC CTC CGT GAG AGC ..3'
Changes in mutant [3]	**Lys**<br>**A**AA
Mutagenesis primers (33mers) [4]	5'.. CGA GTG AAG TTT AAC **A**AA GCC CTC CGT GAG AGC ..3'<br>3'.. GCT CAC TTC AAA TTG **T**TT CGG GAG GCA CTC TCG ..5'

I777M substitution in p110δ [1]

Wild type sequence [2]	772 **777** 783<br>N'.. Gly Ser Val Gly Ile **Ile** Phe Lys Asn Gly Asp Asp .. C'<br>5'.. GC AGC GTG GGC ATC AT**C** TTT AAG AAC GGG GAT G .. 3'
Changes in mutant [3]	**Met**<br>AT**G**
Mutagenesis primers (33mers) [4]	5'.. GC AGC GTG GGC ATC AT**G** TTT AAG AAC GGG GAT G .. 3'<br>3'.. CG TCG CAC CGC TAG TA**C** AAA TTC TTG CCC CTA C .. 5'

I825V substitution in p110δ [1]

Wild type sequence [2]	820 **825** 829<br>N'.. Asp Arg Thr Gly Leu **Ile** Glu Val Val Leu .. C'<br>5'.. AC CGC ACA GGC CTC **A**TT GAG GTG GTA CTC .. 3'
Changes in mutant [3]	**Val**<br>**G**TT
Mutagenesis primers (26mers) [4]	5'.. AC CGC ACA GGC CTC **G**TT GAG GTG GTA CTC .. 3'<br>3'.. TG GCG TGT CCG GAG **G**AA CTC CAC CAT GAG .. 5'

I800M substitution in p110α [1]

Wild type sequence [2]	800<br>N'..Leu Phe Gln Asn Asn Glu Ile **Ile** Phe Lys Asn Gly Asp Asp Leu Arg..C'<br>5'..CTG TTT CAG AAC AAT GAG ATC AT**C** TTT AAA AAT GGG GAT GAT TTA CG ..3'
Changes in mutant [3]	**Met**<br>AT**G**
Mutagenesis primers (47mers) [4]	**5'..CTG TTT CAG AAC AAT GAG ATC ATC TTT AAA AAT GGG GAT GAT TTA CG..3'**<br>**3´..GAC AAA GTC TTG TTA CTC TAG TAG AAA TTT TTA CCC CTA CTA AAT GC..5'**

Primers for verifying recombinant retrovirus integration

Forward primer (p110δ)	601<br>N'.. Arg Lys Leu Thr Asp Asp Glu ..C'<br>**5'.. CGG AAA CTG ACG GAC GAT GA ..3'**
Reverse primer (vector)	3'.. AAA CGC ACA CCG GTT CCA TT ..5'

[1]: Derivation of mutagenesis primers from coding sequences.

[2]: Amino acid position, protein sequence and coding strand sequence. The amino acids to be substituted are shown in bold print.

[3]: Changed amino acid and codon. Alterations introduced by site-directed mutagenesis are colored blue.

[4]: Forward and reverse primers. Deviations from the wt sequence in the mutagenesis primers are printed in bold and blue and underlined.

Suppl. Table 7: **Antibodies used for signaling analyses.**

**Specificity**	**Species**	**Supplier (specification)**
anti-total Akt	mouse	Cell signaling (C67E7)
anti-haemagglutinin	mouse	Antikörper online
anti-actin	mouse	Merck Millipore (clone C4)
anti-p110δ	rabbit	Cell signaling (D1Q7R)
anti-p110α	rabbit	Cell signaling (C73F8)
anti-phospho-Akt (Ser-473)	rabbit	Cell signaling (D9E)
anti-GAPDH	rabbit	Merck Millipore (mAbE606)
anti-mouse IgG (IRDye 800CW)	goat	Li-Cor Biosciences
anti-rabbit IgG (IRDye 680)	goat	Li-Cor Biosciences

Supplementary References (referring to *Supplementary Tables 1 and 4*)

1. So, L. et al. Selective inhibition of phosphoinositide 3-kinase p110alpha preserves lymphocyte function. *J Biol Chem* **288**, 5718-31 (2013).
2. Fritsch, C. et al. Characterization of the novel and specific PI3Kalpha inhibitor NVP-BYL719 and development of the patient stratification strategy for clinical trials. *Mol Cancer Ther* **13**, 1117-29 (2014).
3. Deng, C. et al. Silencing c-Myc translation as a therapeutic strategy through targeting PI3Kdelta and CK1epsilon in hematological malignancies. *Blood* **129**, 88-99 (2017).
4. Lannutti, B.J. et al. CAL-101, a p110delta selective phosphatidylinositol-3-kinase inhibitor for the treatment of B-cell malignancies, inhibits PI3K signaling and cellular viability. *Blood* **117**, 591-4 (2011).
5. Hoegenauer, K. et al. Discovery of CDZ173 (Leniolisib), Representing a Structurally Novel Class of PI3K Delta-Selective Inhibitors. *ACS Med Chem Lett* **8**, 975-980 (2017).
6. Winkler, D.G. et al. PI3K-delta and PI3K-gamma inhibition by IPI-145 abrogates immune responses and suppresses activity in autoimmune and inflammatory disease models. *Chem Biol* **20**, 1364-74 (2013).
7. Cushing, T.D. et al. Discovery and in vivo evaluation of (S)-N-(1-(7-fluoro-2-(pyridin-2-yl)quinolin-3-yl)ethyl)-9H-purin-6-amine (AMG319) and related PI3Kdelta inhibitors for inflammation and autoimmune disease. *J Med Chem* **58**, 480-511 (2015).
8. Doukas, J. et al. Phosphoinositide 3-kinase gamma/delta inhibition limits infarct size after myocardial ischemia/reperfusion injury. *Proc Natl Acad Sci U S A* **103**, 19866-71 (2006).
9. Evans, C.A. et al. Discovery of a Selective Phosphoinositide-3-Kinase (PI3K)-gamma Inhibitor (IPI-549) as an Immuno-Oncology Clinical Candidate. *ACS Med Chem Lett* **7**, 862-7 (2016).
10. Bell, K. et al. SAR studies around a series of triazolopyridines as potent and selective PI3Kgamma inhibitors. *Bioorg Med Chem Lett* **22**, 5257-63 (2012).
11. Camps, M. et al. Blockade of PI3Kgamma suppresses joint inflammation and damage in mouse models of rheumatoid arthritis. *Nat Med* **11**, 936-43 (2005).
12. Kong, D. & Yamori, T. ZSTK474 is an ATP-competitive inhibitor of class I phosphatidylinositol 3 kinase isoforms. *Cancer Sci* **98**, 1638-42 (2007).

13. Tanaka, H. et al. The selective class I PI3K inhibitor CH5132799 targets human cancers harboring oncogenic PIK3CA mutations. *Clin Cancer Res* **17**, 3272-81 (2011).
14. Knight, Z.A. et al. A pharmacological map of the PI3-K family defines a role for p110alpha in insulin signaling. *Cell* **125**, 733-47 (2006).
15. Ndubaku, C.O. et al. Discovery of 2-{3-[2-(1-isopropyl-3-methyl-1H-1,2-4-triazol-5-yl)-5,6-dihydrobenzo[f]imidazo[1,2-d][1,4]oxazepin-9-yl]-1H-pyrazol-1-yl}-2-methyl-propanamide (GDC-0032): a beta-sparing phosphoinositide 3-kinase inhibitor with high unbound exposure and robust in vivo antitumor activity. *J Med Chem* **56**, 4597-610 (2013).
16. Qian, C. et al. Cancer network disruption by a single molecule inhibitor targeting both histone deacetylase activity and phosphatidylinositol 3-kinase signaling. *Clin Cancer Res* **18**, 4104-13 (2012).
17. Walls, M. et al. Targeting small cell lung cancer harboring PIK3CA mutation with a selective oral PI3K inhibitor PF-4989216. *Clin Cancer Res* **20**, 631-43 (2013).
18. Foster, P. et al. The Selective PI3K Inhibitor XL147 (SAR245408) Inhibits Tumor Growth and Survival and Potentiates the Activity of Chemotherapeutic Agents in Preclinical Tumor Models. *Mol Cancer Ther* **14**, 931-40 (2015).
19. Liu, N. et al. BAY 80-6946 is a highly selective intravenous PI3K inhibitor with potent p110alpha and p110delta activities in tumor cell lines and xenograft models. *Mol Cancer Ther* **12**, 2319-30 (2013).
20. Yuan, J. et al. PF-04691502, a potent and selective oral inhibitor of PI3K and mTOR kinases with antitumor activity. *Mol Cancer Ther* **10**, 2189-99 (2012).
21. Barlaam, B. et al. Discovery of 9-(1-anilinoethyl)-2-morpholino-4-oxo-pyrido[1,2-a]pyrimidine-7-carboxamides as PI3Kbeta/delta inhibitors for the treatment of PTEN-deficient tumours. *Bioorg Med Chem Lett* **24**, 3928-35 (2015).
22. Folkes, A.J. et al. The identification of 2-(1H-indazol-4-yl)-6-(4-methanesulfonyl-piperazin-1-ylmethyl)-4-morpholin-4-yl-t hieno[3,2-d]pyrimidine (GDC-0941) as a potent, selective, orally bioavailable inhibitor of class I PI3 kinase for the treatment of cancer. *J Med Chem* **51**, 5522-32 (2008).
23. Beaufils, F. et al. 5-(4,6-Dimorpholino-1,3,5-triazin-2-yl)-4-(trifluoromethyl)pyridin-2-amine (PQR309), a Potent, Brain-Penetrant, Orally Bioavailable, Pan-Class I PI3K/mTOR Inhibitor as Clinical Candidate in Oncology. *J Med Chem* **60**, 7524-7538 (2017).

24. Markman, B. et al. Phase I safety, pharmacokinetic, and pharmacodynamic study of the oral phosphatidylinositol-3-kinase and mTOR inhibitor BGT226 in patients with advanced solid tumors. *Ann Oncol* **23**, 2399-408 (2012).
25. Yu, P. et al. Characterization of the activity of the PI3K/mTOR inhibitor XL765 (SAR245409) in tumor models with diverse genetic alterations affecting the PI3K pathway. *Mol Cancer Ther* **13**, 1078-91 (2014).
26. Maira, S.M. et al. Identification and characterization of NVP-BKM120, an orally available pan-class I PI3-kinase inhibitor. *Mol Cancer Ther* **11**, 317-28 (2011).
27. Sutherlin, D.P. et al. Discovery of a potent, selective, and orally available class I phosphatidylinositol 3-kinase (PI3K)/mammalian target of rapamycin (mTOR) kinase inhibitor (GDC-0980) for the treatment of cancer. *J Med Chem* **54**, 7579-87 (2011).
28. Maira, S.M. et al. Identification and characterization of NVP-BEZ235, a new orally available dual phosphatidylinositol 3-kinase/mammalian target of rapamycin inhibitor with potent in vivo antitumor activity. *Mol Cancer Ther* **7**, 1851-63 (2008).
29. Rewcastle, G.W. et al. Biological characterization of SN32976, a selective inhibitor of PI3K and mTOR with preferential activity to PI3Kalpha, in comparison to established pan PI3K inhibitors. *Oncotarget* **8**, 47725-47740 (2017).
30. Hart, S. et al. VS-5584, a novel and highly selective PI3K/mTOR kinase inhibitor for the treatment of cancer. *Mol Cancer Ther* **12**, 151-61 (2012).
31. Somoza, J.R. et al. Structural, biochemical, and biophysical characterization of idelalisib binding to phosphoinositide 3-kinase delta. *J Biol Chem* **290**, 8439-46 (2015).
32. Berndt, A. et al. The p110 delta structure: mechanisms for selectivity and potency of new PI(3)K inhibitors. *Nat Chem Biol* **6**, 117-24 (2010).
33. Walker, E.H. et al. Structural determinants of phosphoinositide 3-kinase inhibition by wortmannin, LY294002, quercetin, myricetin, and staurosporine. *Mol Cell* **6**, 909-19 (2000).
34. Zhao, Y. et al. Crystal structures of PI3Kalpha complexed with PI103 and its derivatives: new directions for inhibitors design. *ACS Med Chem Lett* **5**, 138-42 (2013).
35. Knight, S.D. et al. Discovery of GSK2126458, a highly potent inhibitor of PI3K and the mammalian target of rapamycin. *ACS Med Chem Lett* **1**, 39-43 (2010).
36. Cheng, H. et al. Discovery of the highly potent PI3K/mTOR dual inhibitor PF-04979064 through structure-based drug design. *ACS Med Chem Lett* **4**, 91-7 (2013).

37. Scott, W.J. et al. Discovery and SAR of novel 2,3-dihydroimidazo[1,2-c]quinazoline PI3K inhibitors: identification of copanlisib (BAY 80-6946). *ChemMedChem* **11**, 1517-30 (2016).

38. Castanedo, G.M. et al. Structure-based design of tricyclic NF-kappaB inducing kinase (NIK) inhibitors that have high selectivity over phosphoinositide-3-kinase (PI3K). *J Med Chem* **60**, 627-640 (2017).
