# Supplementary material for: Functional impact and molecular binding modes of drugs that target the PI3K isoform p110δ
Source: Commun Biol. 2023 Jun 5;6:603. doi: 10.1038/s42003-023-04921-z (PMC10241892; doi:10.1038/s42003-023-04921-z)
Supplement: Supplementary file 3 — Description of Additional Supplementary Files [file 42003_2023_4921_MOESM3_ESM.pdf]

**Description of Additional Supplementary Files**

**File name:** Supplementary Movie 1

**Description:** Physical movements of the specificity pocket residues M752 and W760 and of the mutated residue 777 in the apo forms of p110δ-wt and p110δ-I777M. The movie shows higher flexibility of side chains in mutant p110δ (right) than in wild type p110δ (left). Of note, the specificity pocket is not formed in these simulations in the absence of inhibitor.

**File name:** Supplementary Movie 2

**Description***:* Interaction of ATP-binding pocket residues M752, W760 and I/M777 with idelalisib. In p110δ-wt (left), the specificity pocket formed by M752 and W760 accommodates idelalisib, whereas its formation is prevented in p110δ-I777M (right).

**File name:** Supplementary Movie 3

**Description***:* Interaction of ZSTK474 with residues I777 and K779 of p110δ. Comparisons of side chain movements in the apo (black mesh) and ZSTK474-bound (green mesh) p110δ-wt suggest an induced fit of ZSTK474 between residues I777 and K779.

**File name:** Supplementary Movie 4

**Description:** Binding mode of ZSTK474 to p110δ-wt. ZSTK474 is accommodated between the N-lobe (dark grey) and C-lobe (light grey) of p110δ-wt. Residues important for ZSTK474 binding are shown as sticks.

**File name:** Supplementary Movie 5

**Description***:* Interaction of copanlisib with p110δ-I777M. Copanlisib is accommodated between the N-lobe (dark grey) and C-lobe (light grey) of p110δ-I777M. Residues important for copanlisib binding are colored purple in the surface view and shown as sticks.

**File name:** Supplementary Data 1

**Description:** Structure coordinates in pdb format; input and output files as well as representative structures.

**File name:** Supplementary Data 2

**Description:** Source data for graphs in the paper.
